# Multimode Fiber-Tip Interferometry for Time- and Frequency-Domain Analysis of Droplet Evaporation

**DOI:** 10.3390/s26185742

**Published:** 2026-09-09

**Authors:** Mário Lousada, Vinícius Piaia, Paulo Robalinho, Susana Silva, Susana Novais, Orlando Frazão

**Affiliations:** 1 INESC TEC—Institute for Systems and Computer Engineering, Technology and Science, Rua Dr. Roberto Frias, 4200-465 Porto, Portugal; mario.r.alves@inesctec.pt (M.L.); vinicius.piaia@inesctec.pt (V.P.); paulo.robalinho@inesctec.pt (P.R.); susana.o.silva@inesctec.pt (S.S.); orlando.frazao@inesctec.pt (O.F.); 2 FEUP—Faculty of Engineering of the University of Porto, Rua Dr. Roberto Frias, 4200-465 Porto, Portugal

**Keywords:** multimode optical fiber, fiber-tip interferometer, droplet evaporation, thickness variation, fast Fourier transform, Hilbert transform, binary mixtures

## Abstract

This work presents an experimental investigation of droplet evaporation dynamics using a step-index multimode fiber-tip (MMF) interferometer. Distilled water, ethanol, isopropyl alcohol (IPA), and their binary mixtures with water were analyzed through complementary frequency- and time-domain approaches. Fast Fourier Transform (FFT) analysis was used to identify the dominant spectral components over selected evaporation intervals, while the instantaneous phase obtained from the analytic signal was used to track time-dependent variations in the optical response. For water, dominant components at 8.34 and 9.87 Hz corresponded to thickness-variation rates of −4.85 and −5.75 µm/s, respectively. Ethanol exhibited a dominant component at 15.8 Hz, corresponding to −9.01 µm/s, whereas IPA showed components at 13.2 and 34.8 Hz, associated with rates of −7.46 and −19.6 µm/s. Binary mixtures exhibited multiple spectral components and stronger temporal variability, indicating a nonstationary optical response during evaporation. The frequency- and time-domain results therefore provide complementary descriptions: the FFT identifies the dominant components over the selected interval, whereas instantaneous-phase analysis reveals their temporal evolution. Because the analysis was performed over short, selected evaporation intervals, the refractive index was assumed to remain approximately constant, and the measured phase variations were therefore attributed predominantly to changes in droplet thickness. The retrieved values are consequently interpreted as thickness-variation rates rather than direct mass-loss rates. The proposed approach provides a simple and compact method for monitoring droplet evaporation.

## 1. Introduction

Evaporation is the phase-change process in which a liquid transitions into vapor. The evaporation of liquid droplets has attracted sustained attention across the life, health, and physical sciences, with the number of related publications increasing by about one order of magnitude over the last 50 years [1]. Beyond its scientific relevance, evaporation dynamics is central to several industrial applications, including electronic circuit cooling [2], liquid combustion in automotive engines [3], inkjet printing [4] and even in the food and beverage industry [5].

The monitoring of liquid evaporation and the investigation of the associated physical and chemical properties have therefore been extensively studied over the years. Droplet evaporation is governed by a combination of intrinsic material properties, such as concentration, viscosity, surface tension, thermal conductivity, and mass diffusivity [6], as well as extrinsic environmental conditions, including temperature, relative humidity, and pressure [7]. This interplay leads to complex behavior and motivates the development of models that incorporate multiple coupled parameters.

To investigate these dynamics, a variety of experimental techniques have been employed. Among the currently available approaches, image-based methods are widely used, since the analysis of droplet profiles can provide relevant information about evaporation behavior [1,8,9]. Other techniques have also been applied, including infrared spectroscopy [10], thermography [7], gas chromatography [11], and ion flow tube mass spectrometry [12]. In addition, methods such as particle image velocimetry [13], spectral-radar optical coherence tomography [14], high-speed confocal microscopy [15], aggregation-induced emission luminogens [16], and cavity-enhanced Raman scattering combined with laser-induced fluorescence [17] have also been explored to obtain a deeper understanding of evaporation dynamics.

Although the methods are highly reliable for detecting evaporation dynamics, they often require complex models, expensive equipment, and extensive calibration. In this context, optical fiber sensors have emerged as an attractive alternative, offering high resolution, fast response, and reduced susceptibility to drift [18]. For more applied scenarios, it is often sufficient to determine the evaporation rate to identify the liquid. Therefore, optical fiber-based methods have been increasingly developed due to their lower cost and simpler data processing.

The simplest fiber-optic configuration consists of a single-mode fiber (SMF) with a droplet placed at its tip, producing a Fabry–Pérot-like response through reflections at the fiber–liquid and liquid–air interfaces [19]. Based on this principle, fiber-tip interferometric approaches have been combined with signal-processing techniques such as FFT and Hilbert-transform analysis to retrieve the temporal evolution of liquid evaporation [20], while data-driven methods have also been explored for liquid identification from fiber-tip signals [21]. Other configurations have also been investigated, including microcell cavities formed between two SMF sections [22], a hollow square-core fiber arranged as a Mach–Zehnder interferometer [23], whispering-gallery cavities formed during droplet evaporation [24], and reflective sensors based on microstructured [25] and suspended-core fibers [26]. More recently, compact fiber-optic interferometers and dynamic Fabry–Pérot microcavities have been proposed for monitoring evaporation dynamics and liquid composition [27,28]. These studies demonstrate the potential of fiber-optic interferometry for evaporation monitoring; however, most reported approaches rely on single-mode or specialty-fiber configurations, and the temporal evolution of the composite spectral response generated by a step-index MMF tip remains insufficiently investigated.

Previous fiber-optic approaches to droplet evaporation have used different sensing and signal-processing strategies. Lim and Ahn employed a single-mode fiber-tip interferometer combined with FFT and Hilbert-transform analysis to monitor evaporation dynamics, whereas Wassana et al. used machine-learning techniques to identify liquids from fiber-tip signals. Pereira et al. investigated ethanol–water evaporation using a specialty hollow square-core fiber configured as a Mach–Zehnder interferometer. In contrast, the present work employs a short section of conventional step-index multimode fiber in a reflective fiber-tip configuration and focuses on the combined frequency- and time-domain response produced by the multimode interferometric field during droplet evaporation.

Building on this distinction, the short step-index MMF section is used as a reflective fiber-tip interferometer to investigate the evaporation dynamics of distilled water, ethanol, IPA, and their binary mixtures with water.

The FFT and Hilbert transform are used as complementary analysis tools: the FFT provides high spectral resolution for identifying the dominant interference components within selected evaporation intervals, while the phase of the analytic signal enables direct tracking of their temporal evolution. Sliding-window spectral methods were not adopted because the emphasis of this study is on spectral resolution and accurate phase tracking rather than real-time processing, thereby avoiding the resolution loss and temporal averaging associated with finite sliding windows. The study focuses on the complementary interpretation of the frequency- and time-domain responses and on the influence of liquid composition on the resulting interference signatures. Because the analysis is performed over short, selected evaporation intervals, the refractive index is assumed to remain approximately constant during each analyzed interval. Under this approximation, the measured phase variations are attributed predominantly to changes in droplet thickness, and the extracted values are interpreted as thickness-variation rates. The resulting features are evaluated as descriptors for discriminating among the liquid compositions investigated in this study. The main novelty of the proposed method lies in its simple strategy for material differentiation, directly exploiting the distinct time-dependent interferometric response produced by each material.

## 2. Operating Principle and Signal Analysis

**Figure 1:**
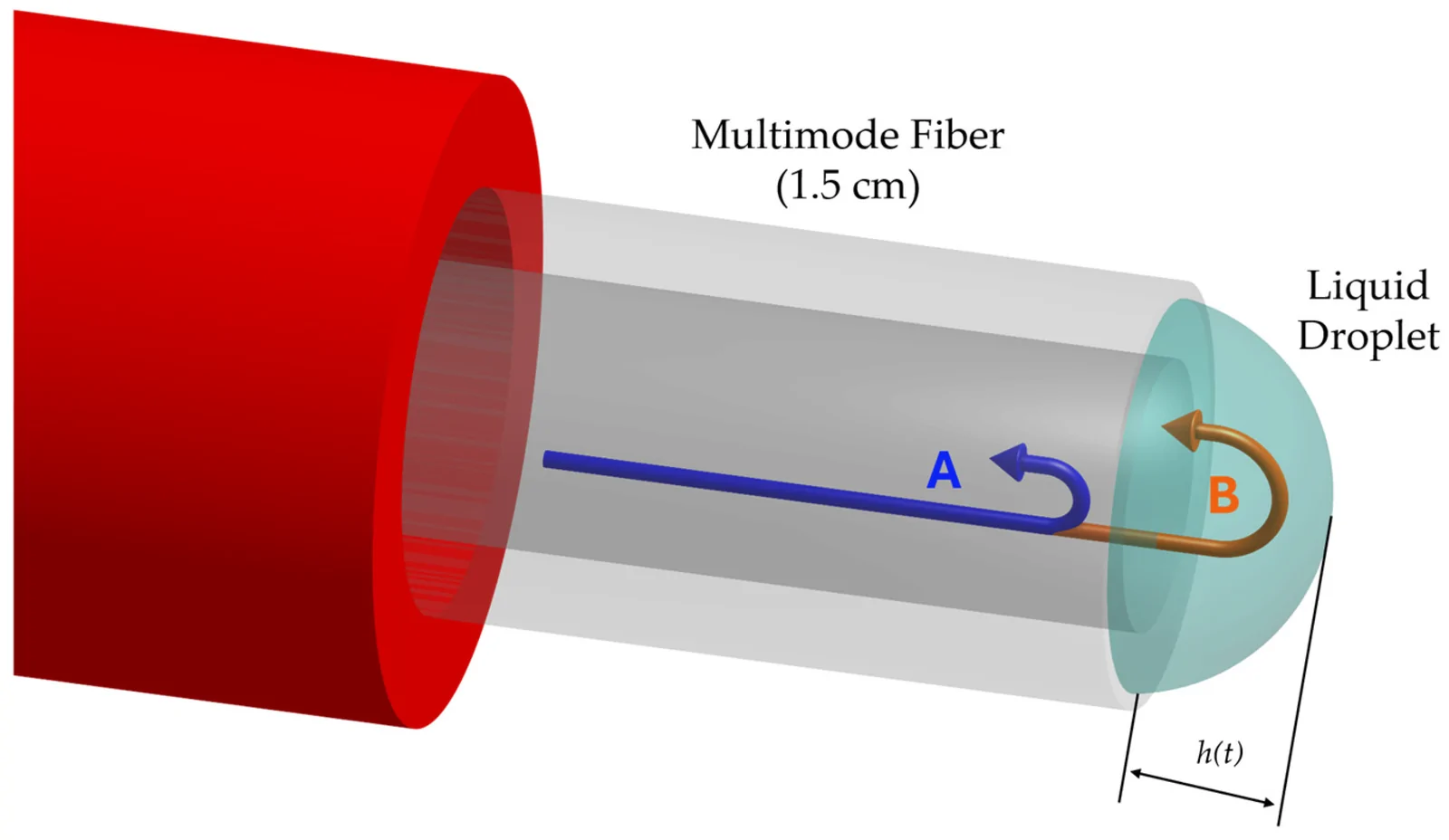
Schematic representation of the interferometric cavity at the fiber tip. The interferometer consists of two partial reflections of the input light beam: one at the cleaved fiber tip, creating optical path A, and the other at the liquid–air interface within the liquid droplet, creating optical path B. The cavity thickness is given by the droplet thickness, which is time-dependent: *h*(*t*).

As illustrated in Figure 1, when a liquid droplet is formed at the fiber tip, the incident optical field encounters the fiber–liquid interface, where part of the light is reflected and the remaining part is transmitted into the liquid. A second reflection occurs at the liquid–air interface. The two reflected components propagate back through the fiber and interfere at the photodetector. As the droplet evaporates, the optical path length of the liquid cavity changes, producing a time-dependent modulation of the reflected optical power. The droplet can therefore be approximated as a time-varying Fabry–Pérot cavity.

Following the fiber-tip interferometric model reported in [20], the variation in cavity thickness *dh* associated with one complete interference cycle (phase shift of 2π) is expressed as:

(1) $$dh=\frac{\lambda }{2n_{liq}}$$

where λ is the free-space wavelength, and *n_liq_* is the refractive index of the liquid. As the droplet evaporates, the cavity thickness decreases, producing a periodic modulation of the optical power reflected. Under the assumption that the refractive index remains approximately constant, the average thickness variation can be described as:

(2) $$\Omega_{avg}=-\frac{dh}{dt}$$

where *Ω_avg_* represents the average thickness-variation rate and *dt* is the time interval associated with the corresponding thickness change. For one complete interference cycle, the corresponding time interval is dt=1/ν. Assuming a linear thickness variation with time during evaporation, the dynamic response is given by:

(3) $$\Omega_{avg}=-dh\cdot \nu ,$$

where *ν* is the oscillation frequency in Hertz obtained from the FFT analysis. The FFT provides a global representation of the dominant frequency components contained in the selected interval. However, it does not indicate whether these components remain constant throughout the evaporation process or evolve with time.

To obtain a time-resolved description of the thickness variation, the instantaneous phase of the measured signal is determined from its analytic representation. The instantaneous phase, *Φ*(*t*), is calculated as:

(4) $$\Phi \left(t\right)=\arctan\left(\frac{H_{y}}{y}\right)\propto kn_{liq}2h\left(t\right)=\frac{4\pi n_{liq}h\left(t\right)}{\lambda },$$

where *y* is the measured signal, *H_y_* is its Hilbert transform, *k* is the wavenumber and *h*(*t*) is the cavity thickness over the selected time interval. Using this approach, the definition of droplet thickness variation given in Equation (2) can be reformulated as:

(5) $$\Omega \left(t\right)=-\frac{\partial h}{\partial t}\approx -\frac{\lambda }{4\pi n_{liq}}\frac{d\Phi }{dt}$$

Equation (5) provides a time-dependent estimate of the thickness-variation rate, allowing transient changes and deviations from a quasi-steady response to be identified. In contrast, the FFT-derived values characterize the complete selected time interval. Because the analysis is performed over a short, selected evaporation interval, the refractive index is assumed to remain approximately constant and its temporal variation $\frac{\partial n}{\partial t}$ is therefore neglected in the simplified model. For binary mixtures, preferential evaporation may lead to refractive-index changes over longer evaporation times; this effect is not explicitly included in the present simplified model.

In the MMF, several propagation modes with different angles and optical path lengths coexist, resulting in a superposition of multiple interference contributions. Consequently, the reflected signal is not described by a single Fabry–Pérot response, but by the combined effect of several modal interference patterns. This behavior can produce multiple spectral components in the FFT analysis, even for pure liquids. In binary mixtures, the spectral response may become further affected by temporal changes in composition and evaporation behavior. Therefore, multiple FFT peaks may arise from multimodal interference, from a nonstationary droplet response, or from a combination of both effects. These peaks should not be assigned unambiguously to individual evaporation regimes based on frequency-domain analysis alone. Since the multimode interferometric signal may contain multiple simultaneous spectral components, the instantaneous phase derived from the analytic signal represents the temporal evolution of the composite optical response and should not be interpreted as the instantaneous frequency of a single physical evaporation mode.

## 3. Materials and Methods

The fiber-tip sensor was fabricated by fusion-splicing a 1.5 cm segment of step-index MMF (Thorlabs GmbH, Bergkirchen, Germany, model FG105LCA) to a standard single-mode fiber (SMF). The FG105LCA MMF has a 105 μm pure-silica core, a 125 μm cladding diameter, and a 250 μm acrylate coating diameter. This fiber has a numerical aperture of 0.22. It operates over a wavelength range from 400 to 2400 nm and exhibits a maximum attenuation of approximately 8 dB/km at 808 nm. The SMF-28 fiber has a nominal core diameter of approximately 8.2 μm and a cladding diameter of 125 μm. Its mode-field diameter is approximately 9.2 μm at 1310 nm and 10.4 μm at 1550 nm, with a cutoff wavelength below 1260 nm. The maximum attenuation is approximately 0.32 dB/km at 1310 nm and 0.18 dB/km at 1550 nm.

**Figure 2:**
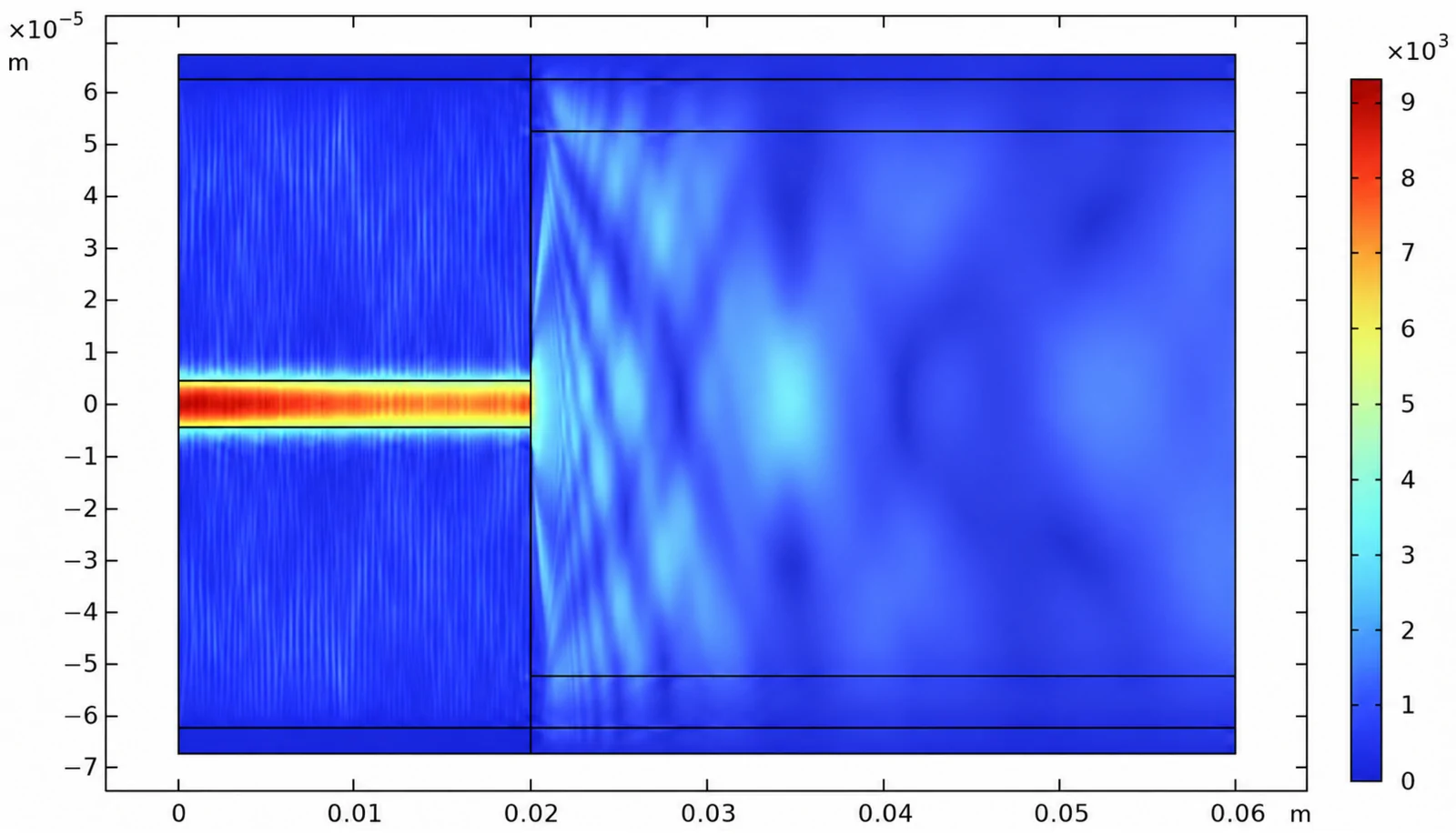
Longitudinal electric-field distribution across the SMF28 to FG105LCA MMF transition simulated using COMSOL Multiphysics.

The longitudinal electric-field distribution across the SMF–MMF transition was simulated using COMSOL Multiphysics^®^ v6.2 (COMSOL AB, Stockholm, Sweden), as shown in Figure 2. In this simulation a 1550 nm wavelength was used. After coupling from the SMF into the larger multimode core, the optical field expands transversely and develops a spatially varying distribution along the MMF section. This pattern is consistent with the excitation and interference of multiple propagation modes. The simulation is intended to illustrate the multimode nature of the optical field within the MMF section and does not establish a direct correspondence between individual propagation modes and the frequency components observed experimentally in the FFT spectra. The selected MMF length places the fiber end within this multimode field distribution, allowing the optical field reaching the tip to interact with the attached liquid droplet.

**Figure 3:**
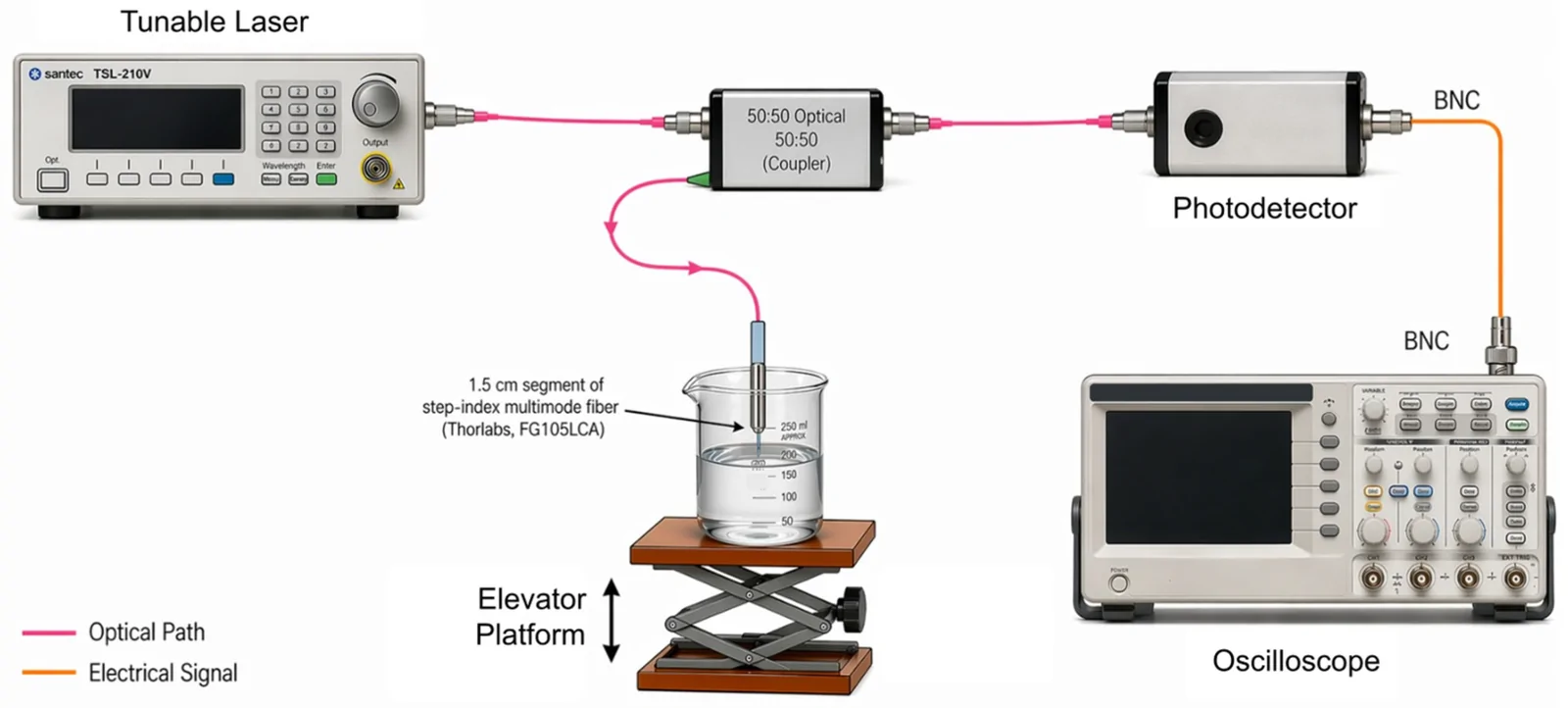
Schematic diagram of the experimental setup, showing the fiber with a liquid droplet at its tip.

The experimental setup is shown in Figure 3. A tunable laser source, Santec TSL-210V (Santec Corporation, Komaki, Japan) was connected to a 50:50 optical coupler, which directed the optical signal toward the fiber-tip sensor and routed the reflected signal to a photodetector (PDA10CS-EC, Thorlabs GmbH, Bergkirchen, Germany). The photodetector output was recorded using a GW Instek GDS-2304A digital oscilloscope (Good Will Instrument Co., Ltd., New Taipei City, Taiwan) at a sampling frequency of 25 kHz. A laboratory jack was used to control the vertical position of the liquid container relative to the fiber tip. The laser wavelength and output power were set to 1550 nm and 8.20 mW, respectively.

A total of nine samples were analyzed: distilled water, ethanol and IPA with nominal purities of 99.8% and 99.5% respectively, and water–alcohol mixtures with nominal alcohol concentrations of 25%, 50%, and 75%. The binary mixtures were prepared from the corresponding alcohol solutions and distilled water and were left to stabilize for approximately 24 h before the measurements.

The refractive index of each sample was measured using an Abbe refractometer (ATAGO DR-A1, Ltd., Tokyo, Japan). The refractive index was measured independently for each of the three experimental repetitions performed for each sample (Table 1 and Table 2). Before each measurement, the fiber tip was carefully cleaned to remove any residual material from the previous experiment and to ensure consistent experimental conditions. The liquid container was then placed on the laboratory jack and raised until the fiber tip was immersed in the sample. The container was subsequently lowered, leaving a droplet attached to the fiber tip.

The complete measurement procedure was repeated three times for each sample. The mean value and standard deviation (SD) were calculated from the three measurements performed for each sample to assess measurement repeatability. Mean values and error bars representing ±1 SD (*n* = 3) were calculated from the individual measurements. The resulting mean values, standard deviations, and relative standard deviations (RSD) are summarized separately for the ethanol and IPA solutions in Table 1 and Table 2, respectively.

Because the laboratory jack was manually operated, small variations in the initial droplet geometry could not be completely avoided. Since the initial droplet volume and geometry were not independently measured for each repetition, the variability specifically introduced by the manual droplet deposition procedure could not be quantitatively determined. The measurements were performed under similar environmental conditions, with the room temperature ranging from 21.4 to 21.7 °C and the relative humidity from 55% to 57%. These parameters were monitored but not actively controlled, and ambient pressure was not independently recorded. Therefore, small environmental variations may have contributed to the variability observed between repeated measurements. Improved control of temperature, humidity, and ambient pressure would further enhance the reproducibility of the proposed method.

For the signal analysis, the evaporation interval corresponding to region (3) was selected because it exhibited the clearest and most approximately periodic interferometric response. Before the FFT analysis, a slowly varying background trend was removed from the selected interval. No additional frequency-selective filtering was applied. No additional tapering window was applied to the selected analysis interval; therefore, its finite duration is equivalent to the use of a rectangular window. The FFT was then calculated over the complete selected interval, and the most prominent local spectral maxima were identified as the dominant frequency components.

For the time-domain analysis, the same interval was used to construct the analytic signal through the Hilbert transform. The instantaneous phase was unwrapped to obtain a continuous phase evolution. The temporal derivative of the phase was evaluated numerically to obtain the thickness-variation rate according to Equation (5).

## 4. Experimental Results and Discussion

**Figure 4:**
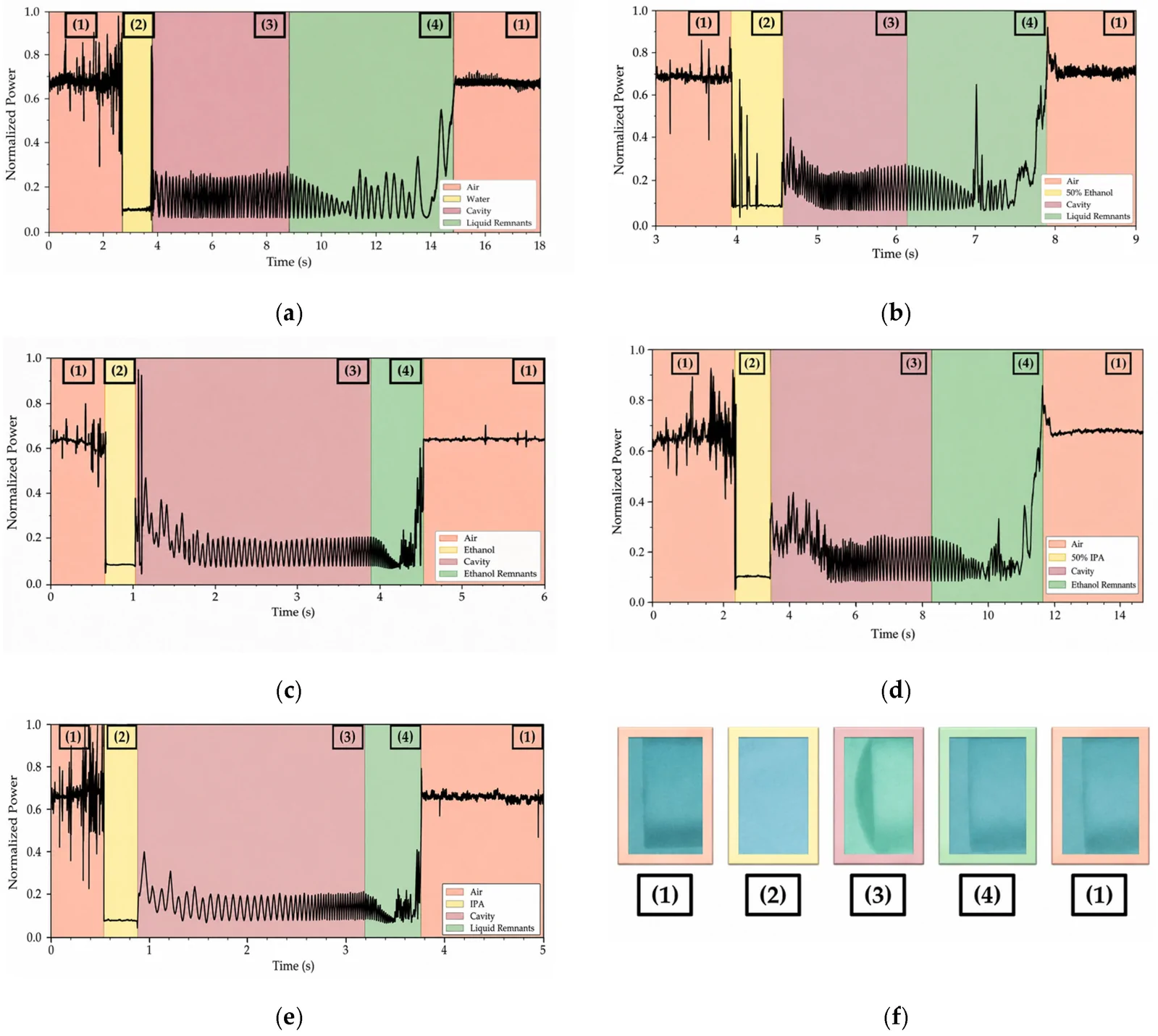
Normalized reflected-power evolution during droplet formation and evaporation for (**a**) distilled water, (**b**) 50% ethanol–water mixture, (**c**) ethanol, (**d**) 50% IPA–water mixture, (**e**) IPA and (**f**) microscope image.

Figure 4 shows the normalized reflected-power evolution obtained for distilled water, ethanol, IPA, and the corresponding 50% water–alcohol mixtures. Although the signal duration and detailed waveform vary among the samples, all measurements exhibit the same general sequence of experimental stages.

Initially, the fiber tip is exposed to air, corresponding to region (1). The tip is then immersed in the liquid, and the reflected signal is mainly determined by the fiber–liquid interface, as indicated by region (2). When the container is lowered, a droplet remains attached to the fiber tip, forming a time-dependent interferometric cavity, corresponding to region (3). During this stage, the reflected optical power exhibits oscillations caused by the variation of the optical path length as the droplet evaporates.

As evaporation proceeds, the oscillation amplitude decreases and the signal becomes smoother, as observed in region (4). This behavior is consistent with a reduction in interference visibility caused by changes in droplet thickness and geometry. After complete evaporation, the reflected power approaches the initial level associated with the fiber tip in air.

For the subsequent FFT and Hilbert-transform analyses, the interval corresponding to region (3) was selected because it contained the clearest and most approximately periodic interferometric response. A slowly varying background trend was removed from this interval before the frequency-domain analysis.

The total duration of the evaporation process observed in this work was considerably shorter than that reported in [20], where evaporation times of approximately 500 s were obtained. In the present experiments, the complete process typically occurred within a few seconds to approximately 18 s, depending on the liquid. This difference is likely influenced by several factors, including the initial droplet volume and geometry, the method used to form the droplet, the fiber-tip dimensions, the physicochemical properties of the liquid, and the surrounding environmental conditions. In the present setup, the droplet was formed by immersing and subsequently withdrawing the fiber tip from the liquid, which is expected to leave a relatively small volume attached to the fiber. However, since the initial droplet volume was not directly measured, its contribution to the shorter evaporation time cannot be quantified independently.

A smaller droplet generally presents a higher surface-area-to-volume ratio, which can enhance mass transfer and contribute to faster evaporation [29]. Nevertheless, the evaporation time should be interpreted as the combined result of droplet geometry, liquid properties, and ambient conditions rather than being attributed to a single parameter.

In addition to the differences in evaporation time, the measured signals exhibit distinct waveform shapes for the analyzed liquids. This behavior is associated with the multimode nature of the sensing section, in which several propagation modes with different optical paths contribute to the reflected signal. The resulting response is therefore a superposition of multiple interference components rather than a single ideal Fabry–Pérot cavity contribution.

This multimodal behavior can lead to the appearance of more than one spectral component, even for a nominally pure liquid. In binary mixtures, the response may become further influenced by droplet geometry, nonstationary evaporation dynamics, and multimode interference. Consequently, multiple FFT peaks should not be interpreted as direct evidence of distinct evaporation regimes without additional temporal analysis.

**Figure 5:**
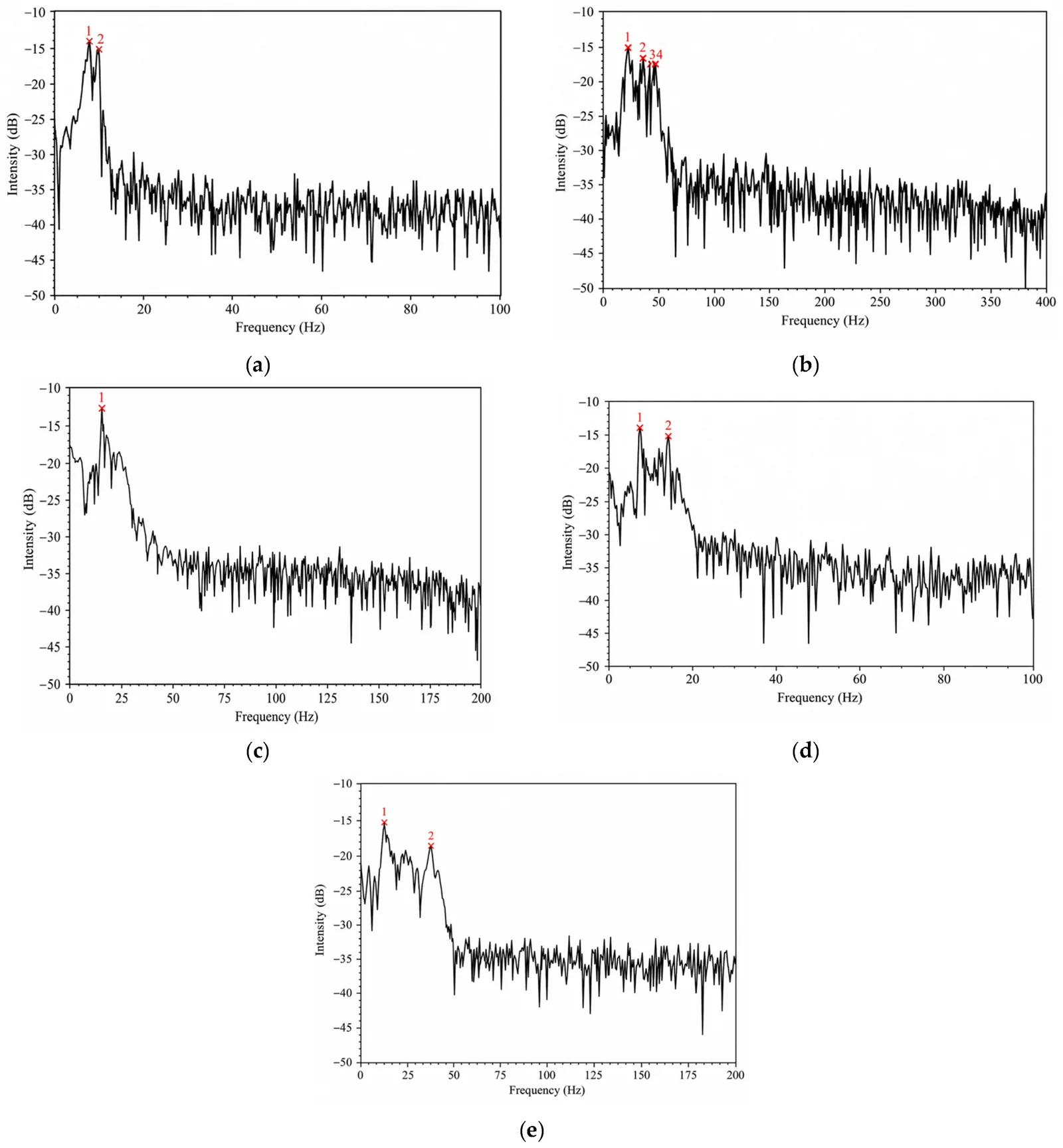
FFT spectra obtained from the selected interferometric interval for (**a**) distilled water, (**b**) 50% ethanol–water mixture, (**c**) ethanol, (**d**) 50% IPA–water mixture, and (**e**) IPA. The red crosses indicate the dominant spectral components used to estimate the thickness-variation rates according to Equation (3).

The FFT spectra obtained from the selected region of the temporal signals are shown in Figure 5. The identified peaks represent the dominant frequency components present over the complete analysis interval. According to Equation (3), these frequencies were converted into effective thickness-variation rates. Higher frequencies correspond to faster changes in the optical path length during the selected interval.

For distilled water (Figure 5a), two dominant spectral components were identified at 8.34 and 9.87 Hz. Using Equation (3), these frequencies correspond to effective thickness-variation rates of −4.85 and −5.75 µm/s, respectively. The relatively low-frequency response is consistent with a slower change in the droplet optical path compared with the alcohol-based samples. The presence of two nearby peaks may arise from the superposition of multimodal interference contributions, small temporal variations during evaporation, or a combination of both effects. Therefore, the two components should not be interpreted as independent evaporation regimes solely from the FFT spectrum.

For the 50% ethanol–water mixture (Figure 5b), four dominant spectral components were identified at 21.2, 32.6, 40.4, and 43.9 Hz. Using a refractive index of 1.356, these frequencies correspond to thickness-variation rates of −12.2, −18.7, −23.1, and −25.2 µm/s, respectively. The presence of several spectral components indicates that the optical response is more complex than that observed for distilled water or ethanol. This behavior may result from the combined influence of multimodal interference, droplet geometry and nonstationary evaporation dynamics during preferential evaporation of ethanol. However, the FFT spectrum alone does not allow each peak to be assigned unambiguously to a specific evaporation stage or liquid component.

For ethanol (Figure 5c), a dominant spectral component was identified at 15.8 Hz, corresponding to a thickness-variation rate of −9.01 µm/s. Compared with distilled water, the higher dominant frequency indicates a faster variation of the droplet optical path during the selected interval. The comparatively simpler spectrum suggests a more uniform response over the analyzed period, although smaller secondary contributions remain visible and may be associated with multimodal interference and transient changes in droplet geometry.

For the 50% IPA–water mixture (Figure 5d), two dominant spectral components were identified at 8.35 and 15.0 Hz. These frequencies indicate the coexistence of slower and faster variations in the droplet optical response during the selected interval. The lower-frequency component is close to that observed for distilled water, while the higher-frequency component reflects a faster variation of the optical path. This behavior may result from the combined effects of multimodal interference and the time-dependent evolution of the binary mixture during evaporation.

Finally, pure IPA (Figure 5e) exhibited two major peaks at 13.2 Hz and 34.8 Hz, corresponding to a thickness-variation rate of −7.46 µm/s and −19.6 µm/s. The presence of multiple components, even in a nominally pure liquid, may be attributed to multimode interference effects and possible transient internal flows or concentration variations within the droplet. A comparison of all spectra in Figure 5 shows that each liquid exhibits a distinct spectral response, effectively acting as a fingerprint. The identification of multiple peaks for water, in contrast to previous reports, further supports the role of multimode interference in shaping the observed response. For the mixtures, the results can be interpreted in the context of previous studies on azeotropic behavior. Water–alcohol mixtures do not evaporate as single-component systems; instead, their composition evolves over time due to preferential evaporation of the more volatile component [9].

Overall, the FFT analysis shows that each tested sample exhibits a distinct distribution of dominant frequency components. Pure liquids generally present simpler spectral responses, whereas the binary mixtures tend to exhibit a larger number of components. Nevertheless, the spectral complexity cannot be attributed exclusively to composition-dependent evaporation, since the multimode nature of the sensor also contributes to the measured response. For this reason, the FFT results are complemented by the instantaneous-phase analysis presented in the following section.

**Figure 6:**
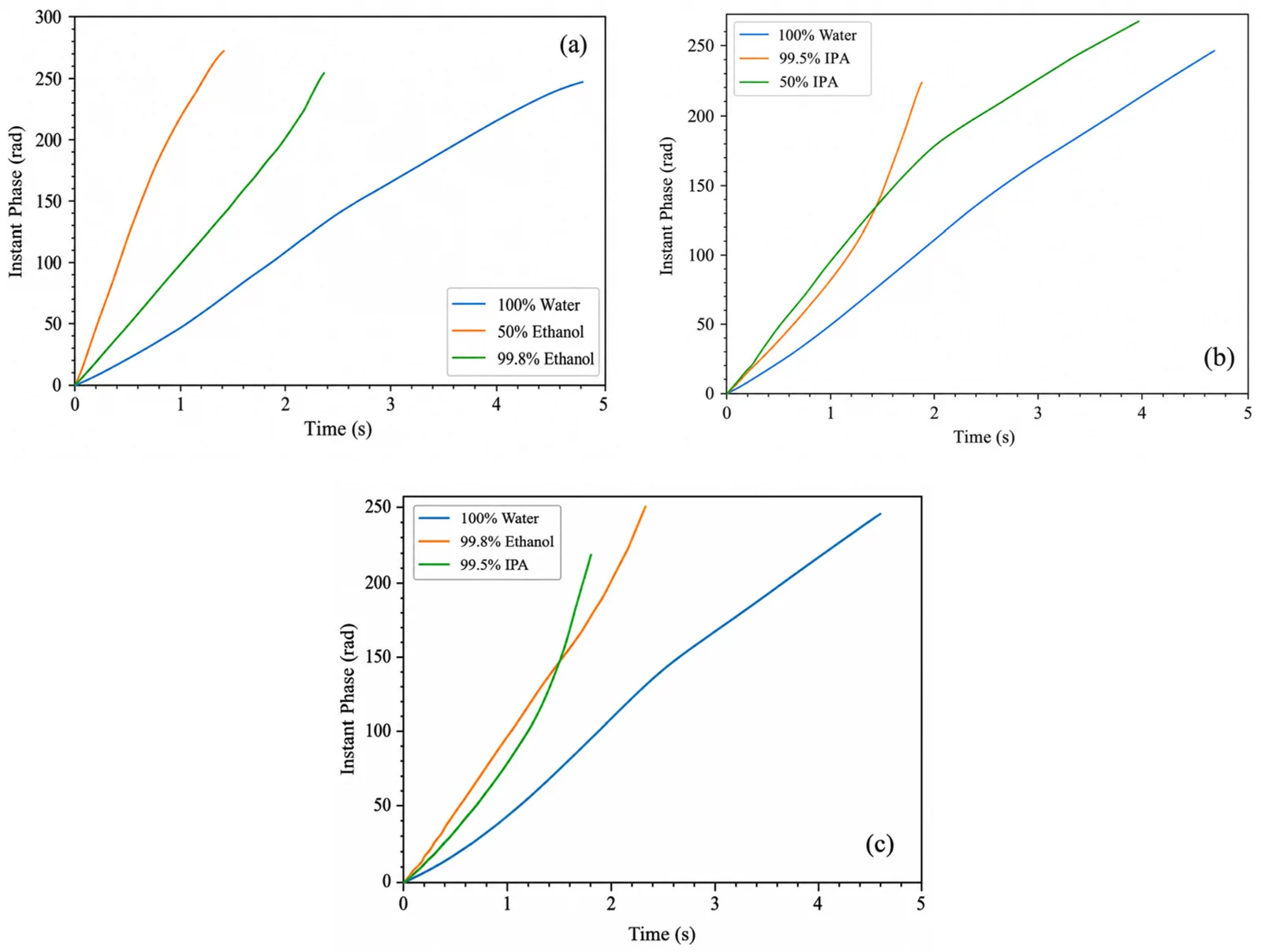
Instantaneous phase evolution for (**a**) water and ethanol-based liquids, (**b**) water and IPA-based liquids, and (**c**) water, ethanol, and IPA.

Figure 6 illustrates the temporal evolution of the phase for the different liquids. The phase evolution reflects the cumulative change in optical path length and, therefore, provides insight into the overall evaporation dynamics.

Approximately linear phase behavior corresponds to a nearly constant optical-path variation rate, as observed for water and, to a lesser extent, for ethanol. In contrast, deviations from linearity in the binary mixtures indicate a nonstationary optical response, which may be associated with changes in droplet geometry, nonstationary evaporation dynamics, and transient effects during evaporation. These results support the use of phase analysis as a complementary tool for investigating the time-dependent behavior of the evaporation process.

**Figure 7:**
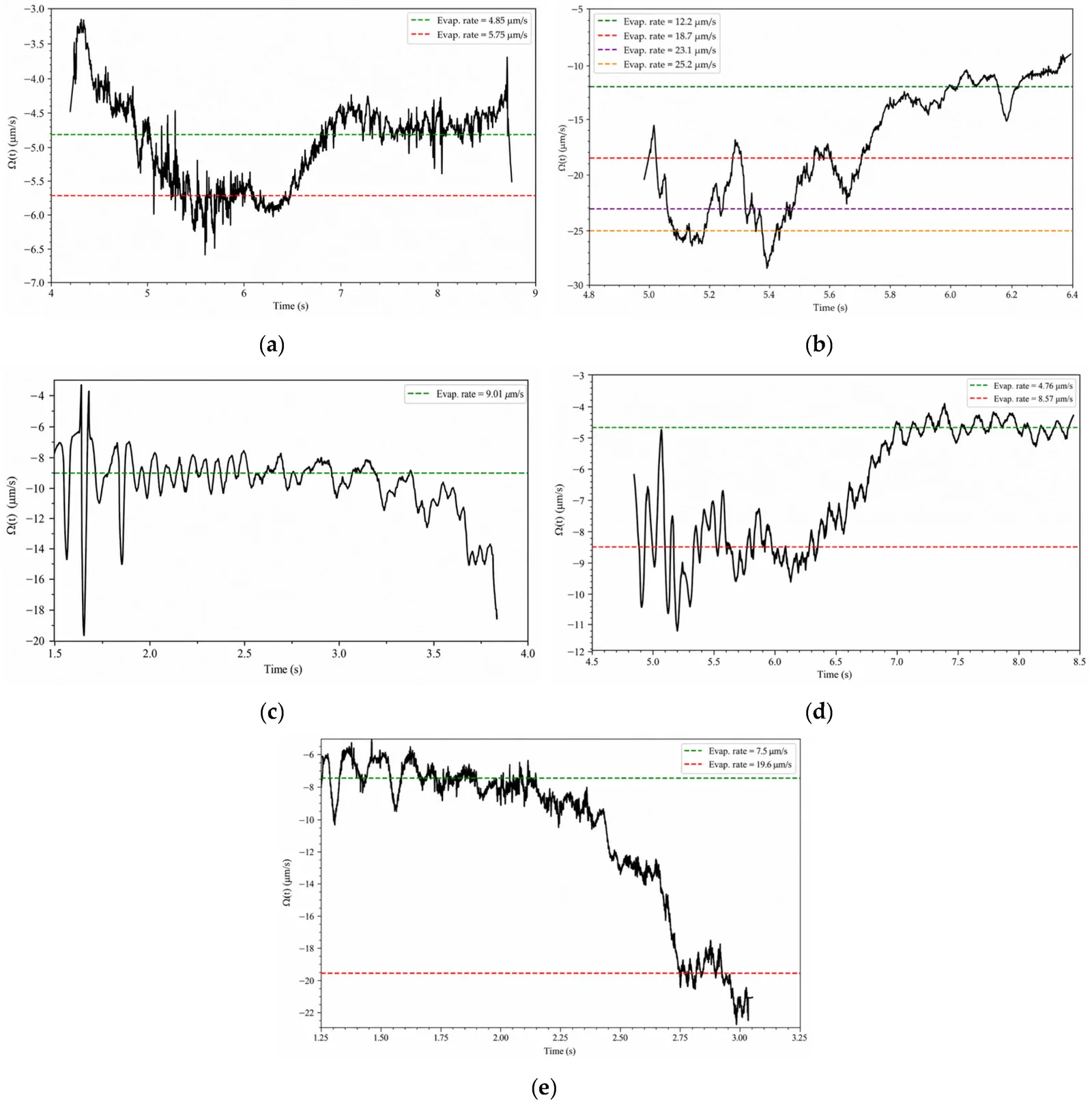
Instantaneous thickness-variation rates obtained for the analyzed liquids using the Hilbert-transform approach. The horizontal dotted lines indicate the corresponding effective rates estimated from the dominant FFT components: (**a**) water, (**b**) 50% ethanol–water mixture, (**c**) ethanol, (**d**) 50% IPA–water mixture, and (**e**) IPA.

Using the same time interval selected for the FFT analysis, the Hilbert transform was applied through Equations (4) and (5), yielding the instantaneous thickness-variation rates shown in Figure 7.

While the FFT provides a global description of the dominant frequency components over the complete analysis interval, the Hilbert-based approach reveals how the optical response evolves with time. This temporal information makes it possible to identify departures from an approximately steady behavior and transient variations that are not visible in the global FFT spectrum.

The fluctuations observed in the instantaneous rate may arise from a combination of physical and signal-processing effects, including changes in droplet geometry, internal fluid motion, multimodal interference, and increased phase sensitivity in intervals of reduced interference visibility. Small mechanical perturbations or transient oscillations of the droplet suspended at the fiber tip may also contribute to local fluctuations in the recorded signal. However, no distinct vibration-dominated component was identified, and the analysis was restricted to the interval showing the clearest and most approximately periodic interferometric response.

Therefore, these fluctuations should be interpreted as variations in the thickness response rather than as direct measurements of the thermodynamic evaporation rate.

For water, Figure 7a shows that the instantaneous thickness-variation rate remains between the two values estimated from the dominant FFT components, with comparatively limited temporal variability over most of the selected interval. The 50% ethanol–water mixture, shown in Figure 7b, exhibits stronger variations and a gradual reduction in the magnitude of the effective rate toward the end of the interval, indicating a more nonstationary optical response.

The 50% IPA–water mixture, shown in Figure 7d, also exhibits pronounced temporal variability. This behavior is consistent with a nonstationary response during the evaporation of binary mixtures, in which preferential evaporation and changes in composition may influence the measured optical signal. Ethanol, shown in Figure 7c, presents a comparatively uniform response over most of the selected intervals. In contrast, IPA, shown in Figure 7e, exhibits a delayed increase in the magnitude of the thickness-variation rate. This behavior may be influenced by the hygroscopic character of the IPA–water system and by competing evaporation and water-vapor absorption processes under humid conditions [30,31]. However, because changes in droplet composition were not independently measured, this interpretation should be regarded as a plausible explanation rather than a definitive assignment.

To evaluate repeatability and the dependence of the optical response on liquid composition, the mean instantaneous thickness-variation rate was calculated for each of the three repeated measurements, and the corresponding absolute values were used for comparison among the tested samples.

Table 1 and Table 2 summarize the mean refractive index and |Ω|(*t*) values, together with the corresponding standard deviations and relative standard deviations (RSD), for ethanol and IPA solutions, respectively. The RSD values ranged from 2.26% to 11.64% for ethanol solutions and from 4.46% to 14.28% for the IPA solutions. These results indicate composition-dependent measurement variability, with the highest dispersion observed for the 99.5% IPA sample.

**Figure 8:**
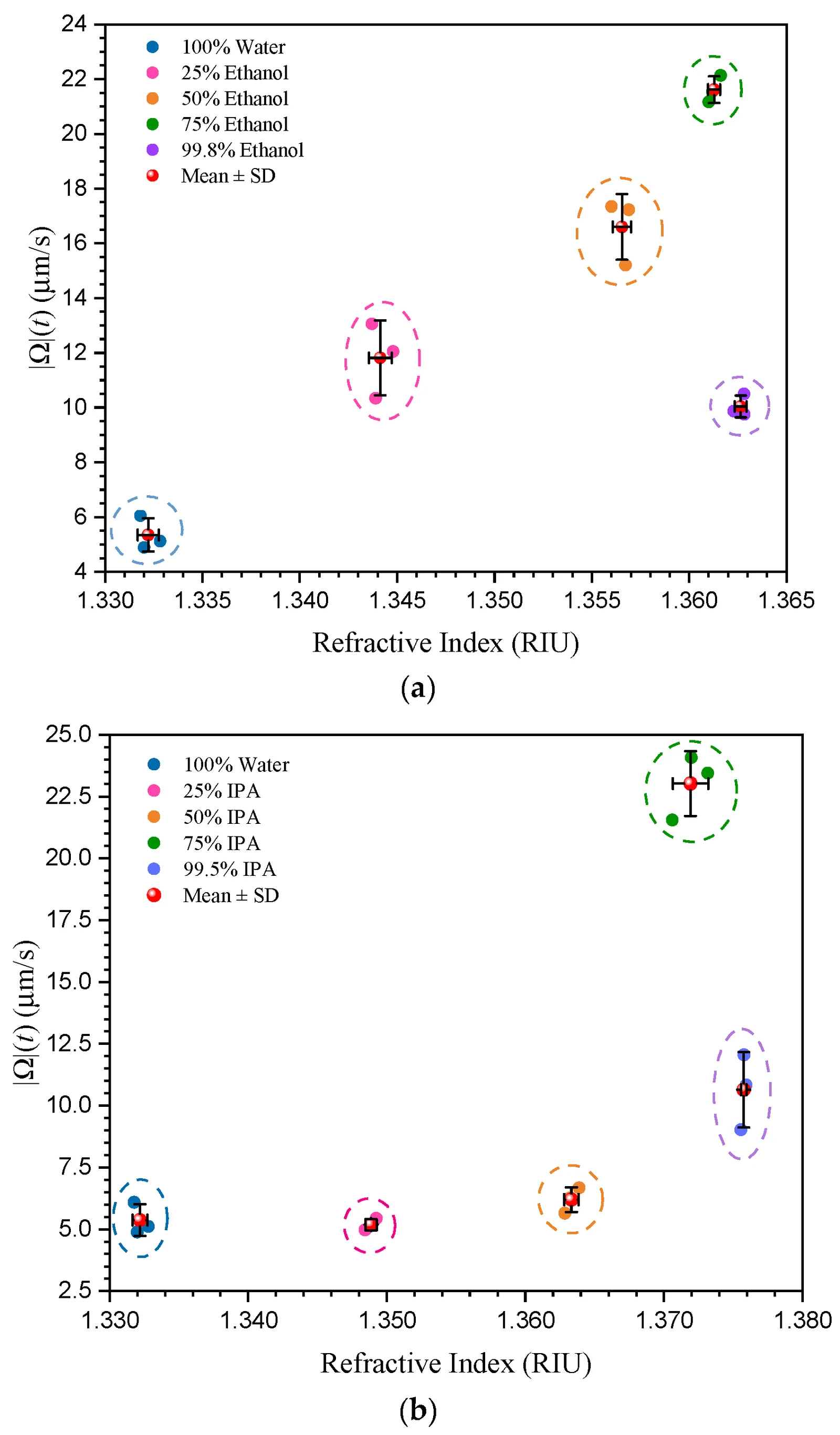
Dependence of ∣Ω∣ (*t*) on refractive index for different concentrations of (**a**) ethanol and (**b**) IPA aqueous solutions. Small markers represent the three individual measurements for each sample, while the larger markers indicate the corresponding mean values. Error bars represent ±1 standard deviation (*n* = 3). (The dashed ellipses are included only as visual guides to group the three repeated measurements for each composition).

For the ethanol-based mixtures, Figure 8a, the effective rate generally increases with ethanol concentration, although the dependence is not strictly monotonic. This behavior may result from the combined influence of volatility, preferential evaporation and differences in droplet geometry. For the IPA-based mixtures, Figure 8b, a different concentration dependence is observed, reflecting the distinct physicochemical behavior of the IPA–water system.

The separation observed among the tested compositions indicates that the extracted effective thickness-variation rate can be used as a descriptive parameter for distinguishing among the samples analyzed in this study. However, considering the limited number of repetitions, these results should be interpreted as evidence of composition-dependent behavior rather than as a general liquid-classification capability.

## 5. Conclusions

This work demonstrated a compact MMF interferometric approach for monitoring the evaporation dynamics of water, ethanol, IPA, and their binary mixtures. The proposed sensor combines frequency-domain and time-domain analyses to characterize variations in the optical path produced during droplet evaporation.

The FFT analysis identified distinct dominant spectral components for the different liquids and mixtures, while the Hilbert-transform approach provided the temporal evolution of the instantaneous thickness-variation rate. The two methods therefore offer complementary information: the FFT describes the dominant behavior over the selected analysis interval, whereas the instantaneous-phase analysis reveals transient and nonstationary variations that are not evident from the global spectrum alone.

The results also showed that the extracted thickness-variation rate depends on liquid composition. Water–ethanol and water–IPA mixtures exhibited different concentration-dependent responses, demonstrating the sensitivity of the interferometric signal to changes in volatility, composition, and droplet dynamics. Under the assumption that the refractive index remains approximately constant over the short, selected analysis intervals, the calculated values are interpreted as thickness-variation rates rather than direct thermodynamic evaporation rates. For binary mixtures, possible refractive-index changes over longer evaporation times remain a limitation of the simplified model.

Despite the limited number of repeated measurements and the use of manually deposited droplets, the experiments showed reproducible composition-dependent trends. These results support the potential of the proposed platform for comparative monitoring of volatile liquids and binary mixtures. Future work should include automated droplet deposition, tighter control of temperature, relative humidity, and ambient pressure, a larger number of repetitions, and simultaneous measurements of droplet mass, geometry, and composition to improve quantitative validation and separate the different physical contributions to the optical response.

## Figures and Tables

**Table 1:** Mean values and standard deviations for the ethanol series.

Sample	Mean RI ± SD (RIU)	Mean |Ω|(t) ± SD (µm/s)	RSD of |Ω|(t) (%)
100% Water	1.3322 ± 0.0005	5.35 ± 0.61	11.47
25% Ethanol	1.3441 ± 0.0006	11.82 ± 1.37	11.64
50% Ethanol	1.3566 ± 0.0005	16.60 ± 1.20	7.24
75% Ethanol	1.3613 ± 0.0003	21.62 ± 0.49	2.26
99.8% Ethanol	1.3627 ± 0.0003	10.04 ± 0.40	4.01

Values are reported as mean ± standard deviation (*n* = 3). RSD = 100 × SD/mean.

**Table 2:** Mean values and standard deviations for the IPA series.

Sample	Mean RI ± SD (RIU)	Mean |Ω|(t) ± SD (µm/s)	RSD of |Ω|(t) (%)
100% Water	1.3322 ± 0.0005	5.37 ± 0.64	11.87
25% IPA	1.3489 ± 0.0004	5.19 ± 0.23	4.46
50% IPA	1.3633 ± 0.0005	6.20 ± 0.52	8.36
75% IPA	1.3719 ± 0.0013	23.02 ± 1.31	5.70
99.5% IPA	1.3758 ± 0.0002	10.64 ± 1.52	14.28

Values are reported as mean ± standard deviation (*n* = 3). RSD = 100 × SD/mean.

## Data Availability

The original contributions presented in this study are included in the article. Further inquiries can be directed to the corresponding author.

## Abbreviations

The following abbreviations are used in this manuscript:

FFT	Fast Fourier Transform
IPA	Isopropyl Alcohol
MMF	Multimode Fiber
RI	Refractive Index
RIU	Refractive Index Units
RSD	Relative Standard Deviation
SD	Standard Deviation
SMF	Single-Mode Fiber
